# Supplementary material for: In silico evaluation of molecular virus–virus interactions taking place between Cotton leaf curl Kokhran virus- Burewala strain and Tomato leaf curl New Delhi virus
Source: PeerJ. 2021 Oct 19;9:e12018. doi: 10.7717/peerj.12018 (PMC8532979; doi:10.7717/peerj.12018)
Supplement: Supplemental Information 2 [file peerj-09-12018-s002.docx]

**Supplementary Table 2:**

Active and Passive Residues of the proteins of CLCuKoV-Bur and ToLCNDV

| **Virus** | **Genes** | **Type of Residues** | **Position of Residues** |
| --- | --- | --- | --- |
| CLCuKoV-Bur | Rep | **Active Residues** | 23, 106, 107, 108, 109, 147, 160, 163, 238, 239, 240, 241, 242, 244, 246,248, 255, 257, 296, 297, 298, 308, 309, 312, 313, 331, 337, 339, 341, |
|  |  | **Passive Residues** | 21, 22, 25, 29, 35, 41, 46, 47, 49, 82, 101, 102, 104, 105,113, 114, 115, 142, 143, 144, 153, 158, 162, 165, 166, 167, 168, 170, 203, 206,209, 210, 211, 212, 214, 217, 228, 231, 232, 235, 236, 237, 250, 251,252,253, 254, 256, 260, 263, 264, 270, 272, 274, 290, 291, 292, 293, 294, 295,304, 305, 306, 307, 315, 316, 320, 321, 323, 324, 325, 326, 329, 330, 333,335, 336, 338, 340, 342, 343, 344, 345, |
|  | TrAP | **Active Residues** | 1, 2, 3, 4, 5, 6, 7, 8, 9, 10, 11, 12, 13, 14, 15,16, 17, 18, 19, 20, 21, 22, 23, 24, 28, 29, 30, 31, 32, 33,34, 35, |
|  |  | **Passive Residues** | 25, 26, 27, |
|  | REn | **Active Residues** | 1, 2, 3, 4, 5, 6, 7, 10, 11, 13, 14, 15, 16, 17, 19,20, 21, 22, 23, 24, 25, 26, 27, 28, 30, 32, 34, 35, 49, 51,53, 54, 57, 58, 60, 61, 62, 63, 64, 65, 66, 70, 71, 72, 74,75, 76, 77, 79, 80, 81, 83, 84, 85, 87, 88, 89, 91, 92, 93,95, 96, 97, 99, 101, 103, 104, 107,110, 111, 114, 115, 119, 121, 122,123, 125, 126, 127, 129, 131, 133, |
|  |  | **Passive Residues** | 8, 36, 37, 38, 39, 40, 41, 42, 43, 45, 55, 59, 68, 78, 118, 120, 124, 128, 134, |
|  | C4 | **Active Residues** | 1, 2, 3, 4, 5, 7, 8, 12, 13, 14, 18, 20, 21, 22, 25,26, 28, 29, 32, 33, 34, 35, 37, 39, 40, 47, 53, 56, 71, 73,74, 81, 82, 83, 90, 101, 104, 106, 108, 110, 113, 114, 115, 116, 118,136, |
|  |  | **Passive Residues** | 15, 16, 17, 36, 38, 41, 42, 48, 49, 51, 52, 54, 55, 57, 58,59, 60, 62, 65, 66, 67, 68, 70, 75, 77, 78, 91, 92, 93, 94,98, 99, 100,103, 111, 112, 121, 122, 124, 125, 126, 127, 128, 131, 132,133, 135, 140, 141, 144, 145, 146, |
|  | CP | **Active Residues** | 1, 5, 6, 17, 18, 19, 20, 23, 25, 26, 28, 29, 30, 32, 34,35, 36, 37, 39, 40, 44, 49, 50, 53, 54, 55, 57, 58, 59, 60,61, 62, 65, 66, 71, 72, 74, 115, 157, 162, 164, 165, 167, 181, 182,201, 202, 249, |
|  |  | **Passive Residues** | 2, 3, 11, 12, 13, 14, 16, 27, 31, 38, 41, 42, 43, 45, 46,47, 48, 51, 52, 63, 64, 67, 75, 76, 85, 98, 99, 100, 101, 102,103, 105, 117, 118, 153, 154, 158, 159, 160, 161, 168, 169, 170, 178, 179,180, 183, 198, 200, 204, 206, 208, 234, 235, 245, 247, 254, 255, |
|  | AV2 | **Active Residues** | 1, 3, 4, 6, 7, 8, 10, 11, 25, 29, 30, 31, 32, 33, 34,35, 36, 37, 39,40, 41, 43, 44, 47, 50, 51, 52, 53, 54, 55,56, 59, 63, 71, 72, 73, 74,101, 113, 115, |
|  |  | **Passive Residues** | 2, 12, 13, 14, 16, 26, 60, 62, 64, 66, 67, 69, 70, 75, 77,78, 79, 82, 84, 90, 91, 94, 95, 96, 97, 98, 99, 100, 102, 103,104, 105, 106, 107, 108, 110, 111, 114, 116, 118, |
| CLCuMB | **βC1** | **Active Residues** | 1, 2, 3, 18, 19, 23, 25, 27, 41, 43, 47, 49, 50, 51, 52,53, 54, 55, 56, 57, 58, 60, 61, 63, 64, 67, 68, 71, 72, 74,75, 76, 79, 84, 85, 86, 89, 92, 93, 96, 97, 103, 104, 105, 106, 107, 108, 109, 110, 112, 115, |
|  |  | **Passive Residues** | 4, 5, 13, 14, 20, 21, 22, 29, 32, 33, 38, 39, 59, 78, 80, 81, 83, 90, 98, 99, 100, 101, 116, 117, 118, |
| ToLCNDV  DNA-A | Rep | **Active Residues** | 10, 43, 44, 46, 47, 48, 50, 54, 57, 58, 91, 155, 156, 158,159,160, 164, 165, 168, 169, 172, 176, 177, 179, 180, 183, 184, 185, 186, 231,234, 235, 258,259, 260, 261, 262, 263, 266, 267, 270, 271, 274, 282, 287,297, 298, 299, 300,301, 303, 311, 313, 314, 315, 317, 318, 321, 322, 325,326, 328, 330, 331, |
|  |  | **Passive Residues** | 1, 2, 3, 4, 5, 6, 7, 8, 9, 11, 12, 13, 34, 39, 40,41, 42, 45, 49, 51, 52, 53, 55, 56, 59, 60, 86, 87, 88, 89,90, 92, 93, 150, 151, 152, 153, 154, 157, 161, 162, 163, 166, 167, 170,171, 173, 174, 175, 178, 181, 182, 187, 188, 189, 190, 212, 227, 228, 230,236, 237, 247, 248, 249, 251, 252, 256, 264, 265, 268, 269, 272, 273, 275, 276, 277, 278, 279, 280, 281, 283, 284, 285, 286, 288, 289, 290, 291, 293,294, 295, 296, 302, 304, 305, 306, 307, 308, 309, 310, 312, 316, 319, 320, 324, 329, 332, 333, 334, 337, 354, 355, 356, 357, |
|  | TrAP | **Active Residues** | 1, 2, 3, 9, 10, 11, 13, 14, 17, 18, 22, 32, 33, 34, 35,36, 37, 38, 39, 40, 46, 47, 49, 50, 51, 54, 55, 57, 58, 59,60, 61, 62, 63, 64, 65, 66, 67, 68, 69, 70, 71, 72, 74, 75,76, 77, 87, 91, 95, 101, 103, 104, 106, 107, 108, 109, 110, 112, 113,114, 116, 117, 118, 119, 120, 122, 123, 124, 125, 126, 127, 128, 130, 131,134, |
|  |  | **Passive Residues** | 4, 12, 15, 19, 21, 23, 24, 25, 26, 27, 28, 29, 30, 31, 45,48, 52, 56, 73, 78, 79, 80, 81, 82, 83, 84, 85, 86, 88, 89,90, 92, 93, 94, 96, 97, 98, 99, 100, 102, 105, 111, 115, 121, 129,132, 133, 135, 136, 137, 139, |
|  | REn | **Active Residues** | 1, 2, 4, 5, 6, 7, 8, 9, 10, 11, 12, 13, 15, 16, 17,18, 19, 21, 22, 23, 24, 25, 26, 27, 28, 30, 32, 34, 38, 39,40, 41, 42, 43, 44, 46, 49, 53, 55, 56, 57, 59, 60, 61, 63,64, 65, 66, 67, 68, 70, 71, 72, 73, 74, 75, 76, 77, 78, 79,80, 81, 82, 83, 85, 86, 87, 89, 90, 91, 93, 94, 95, 97, 98,99, 100, 101, 103, 105, 106, 109, 113, 116, 117, 119, 121, 122, 123, 124,125, 126, 127, 128, 129, 130, 131, 134, 135, |
|  |  | **Passive Residues** | 35, 36, 37, 62, 120, |
|  | C4 | **Active Residues** | 1, 2, 3, 4, 7, 8, 9, 10, 11, 12, 13, 14, 16, 17, 18,19, 20, 21, 22, 23, 24, 25, 27, 28, 29, 30, 31, 32, 33, 35,36, 37, 40, 41, 42, 44, 45, 46, 47, 49, 51, 52, |
|  |  | **Passive Residues** | 15, 38, 43, 48, 50, 53, 54, 55, 56, 57, 58, |
|  | CP | **Active Residues** | 1, 2, 3, 4, 5, 6, 8, 9, 10, 11, 12, 13, 14, 15, 16,18, 19, 20, 21, 22, 23, 24, 25, 26, 27, 28, 30, 31, 32, 33,35, 36, 38, 40, 49, 50, 54, 55, 56, 59, 61, 62, 63, 71, 72,74, 112, 115, 170, 179, 182, 185, 187, 192, 193, 194, 196, 197, 198, 201, 202, 203, 204, 247, 249, |
|  |  | **Passive Residues** | 29, 34, 37, 39, 41, 42, 43, 44, 45, 46, 47, 48, 51, 52, 57,58, 60, 64, 65, 75, 76, 77, 78, 99, 101, 102, 103, 105, 117, 118,119, 121, 124, 127, 128, 129, 130, 131, 132, 134, 136, 141, 157, 167, 168,169, 171, 172, 173, 174, 177, 178,180, 181, 183, 188, 189, 190, 191, 195,200, 205, 206, 234, 245, 254, |
|  | V2 | **Active Residues** | 1, 2, 3, 4, 6, 7, 8, 9, 10, 11, 12, 13, 16, 17, 19, 20, 23, 26, 29, 30, 31, 35, 36, 37, 39, 40, 43, 46, 50, 59,60, 62, 63, 65, 84, 88, 89, 90, 91, 92, 94, 95, 96, 97, 98,105, 106, 107, 108, 109, 110, 111, 112, |
|  |  | **Passive Residues** | 27, 32, 33, 34, 51, 52, 53, 54, 55, 56, 58, 66, 67, 69, 70,71, 77, 78, 79, 80, 82, 83, 93, 99, 100, 101, 102, 103, 104, |
| ToLCNDV  DNA-B | MP | **Active Residues** | 3, 13, 14, 15, 16, 17, 18, 19, 20, 21, 22, 24, 25, 26, 54,55, 61, 63, 64, 65, 67, 68, 69, 70, 91, 92, 100, 115, 116, 117,119, 138, 193, 194, 206, 207, 208, 209, 210, 211, 232, 261, 262, 263, 265, |
|  |  | **Passive Residues** | 1, 2, 5, 6, 8, 10, 11, 12, 23, 27, 28, 31, 36, 37, 42,43, 44, 46, 47, 51, 52, 56, 57, 60, 71, 72, 73, 74, 75, 76,77, 87, 93, 95, 97, 99, 101, 110, 121, 127, 135, 136, 157, 173, 190,196, 203, 204, 205, 229, 230, 231, 233, 235, 250, 257, 259, 260, |
|  | NSP | **Active Residues** | 10, 13, 23, 28, 37, 38, 62, 63, 107, 109, 128, 135, 170, 172, 174,183, 185,188, 191, 192, 195, 196, 197, 206, 207, 211, 242, 244, 245, |
|  |  | **Passive Residues** | 8, 15, 20, 21, 24, 25, 26, 27, 29, 41, 58, 64, 65, 66, 91,92, 93, 95, 97, 103, 104, 105, 106, 108, 110, 111, 121, 123, 125, 126,127, 129, 130, 131, 132, 133, 134, 136, 137, 149, 151, 152, 153, 154, 156,167, 168, 169, 171, 176, 177, 178, 181, 182, 184, 186, 187, 193, 198, 200,203, 204, 212, 213, 214, 227, 228, 229, 238, 241, 243, 246, 247, 248, 249,250, 251, 252, 254, 257, 258, 260, 264, 268, 270, 271, 272, |
