# Supplementary material for: In silico evaluation of molecular virus–virus interactions taking place between Cotton leaf curl Kokhran virus- Burewala strain and Tomato leaf curl New Delhi virus
Source: PeerJ. 2021 Oct 19;9:e12018. doi: 10.7717/peerj.12018 (PMC8532979; doi:10.7717/peerj.12018)
Supplement: Supplemental Information 3 [file peerj-09-12018-s003.docx]

| Intra-virus Protein Interaction | | | Inter-Viral protein interaction | | | Intra-virus Protein Interaction | | |
| --- | --- | --- | --- | --- | --- | --- | --- | --- |
| CLCuKoV/Bu  CLCuMuB | CLCuKoV/Bu  CLCuMuB | HADDOCK SCORE | CLCuKoV/Bu  CLCuMuB | ToLCNDV | HADDOCK SCORE | ToLCNDV DNA-A/ DNA-B | ToLCNDV DNA-A/ DNA-B | HADDOCK SCORE |
| Rep | **Rep** | **-196.8** | Rep | **Rep** | **-201** | Rep | **Rep** | -135.7 |
|  | TrAP | **-154.2** |  | TrAP | **-195.1** |  | TrAP | **-173.1** |
|  | REn | -102.1 |  | REn | -141 |  | Ren | -15 |
|  | C4 | -125.2 |  | C4 | -196 |  | C4 | -89.6 |
|  | CP | -203.9 |  | CP | -192.4 |  | CP | -110.1 |
|  | V2 | **-155.5** |  | V2 | -190.8 |  | V2 | -96.6 |
|  | ßC1 | -136.8 |  | MP | **-279.8** |  | MP | -63.5 |
|  |  |  |  | NSP | **-241.5** |  | NSP | -91.5 |
| TrAP | Rep | -132.7 | TrAP | Rep | -71.6 | TrAP | Rep | -191.8 |
|  | TrAP | -75.7 |  | TrAP | -103.9 |  | TrAP | -120.3 |
|  | REn | -43.7 |  | REn | -58 |  | REn | -44.8 |
|  | C4 | -85.8 |  | C4 | -78.1 |  | C4 | -104.5 |
|  | CP | -75.8 |  | CP | -82.9 |  | CP | **-133.7** |
|  | V2 | -108.2 |  | V2 | -85.9 |  | V2 | -78.0 |
|  | ßC1 | -93.3 |  | MP | -134.9 |  | MP | -73.5 |
|  |  |  |  | NSP | -129.6 |  | NSP | -78.2 |
| REn | Rep | -102.9 | REn | Rep | -58.6 | REn | Rep | -25.6 +/- 21.2 |
|  | TrAP | -17.4 |  | TrAP | -103.2 |  | TrAP | -62.9 +/- 18.0 |
|  | REn | -3.9 |  | REn | 6 |  | REn | 5.9 +/- 21.7 |
|  | C4 | -35.3 |  | C4 | -45.6 |  | C4 | -64.7 +/- 3.7 |
|  | CP | -66.2 |  | CP | -63.7 |  | CP | -17.6 +/- 23.0 |
|  | V2 | -25.4 |  | V2 | -30.3 |  | V2 | -55.6 +/- 13.0 |
|  | ßC1 | -28.9 |  | MP | -109.1 |  | MP | -15.6 +/- 4.8 |
|  |  |  |  | NSP | **-217.8** |  | NSP | -37.9 +/- 13.0 |
| C4 | Rep | -131.2 | C4 | Rep | -65.1 | C4 | Rep | -94.3 +/- 15.9 |
|  | TrAP | -73.6 |  | TrAP | -73.3 |  | TrAP | -118.5 +/- 23.3 |
|  | REn | -39.1 |  | REn | -35.4 |  | REn | -55.3 +/- 11.3 |
|  | C4 | -74.6 |  | C4 | -79.3 |  | C4 | -150.4 +/- 13.2 |
|  | CP | -73.3 |  | CP | -91.9 |  | CP | -133.6 +/- 5.6 |
|  | V2 | -64.9 |  | V2 | -74.4 |  | V2 | -98.4 +/- 15.0 |
|  | ßC1 | -54.9 |  | MP | **-160.9** |  | MP | -115.2 +/- 12.8ac4_m |
|  |  |  |  | NSP | **-129.2** |  | NSP | -112.1 +/- 3.3 |
| CP | Rep | -130.6 | CP | Rep | -100.3 | CP | Rep | -127.9 +/- 16.3 |
|  | TrAP | -74.8 |  | TrAP | -185.3 |  | TrAP | -147.3 +/- 14.4 |
|  | REn | -38.8 |  | REn | 7.8 |  | REn | -58.3 +/- 18.3 |
|  | C4 | -52.9 |  | C4 | -82.5 |  | C4 | -136.7 +/- 2.1 |
|  | CP | -83.6 |  | CP | -163.4 |  | CP | -110.6 +/- 14.5 |
|  | V2 | -94.9 |  | V2 | -64.2 |  | V2 | -119.8 +/- 10.6 |
|  | ßC1 | -93.7 |  | MP | **-166.9** |  | MP | -102.5 +/- 20.2 |
|  |  |  |  | NSP | -110.6 |  | NSP | -87.2 +/- 8.5 |
| V2 | Rep | -149.3 | V2 | Rep | -71.3 | AV2 | Rep | -67.0 +/- 6.2 |
|  | TrAP | -109.9 |  | TrAP | -87.5 |  | TrAP | -83.3 +/- 8.5 |
|  | REn | -48.7 |  | REn | -20.1 |  | REn | -49.1 +/- 12.3 |
|  | C4 | -76.6 |  | C4 | 74.2 |  | C4 | -95.0 +/- 4.2 |
|  | CP | -94.7 |  | CP | -98 |  | CP | -115.7 +/- 1.3a |
|  | V2 | -96.9 |  | AV2 | -78.7 |  | V2 | -48.8 +/- 24.7 |
|  | ßC1 | -66 |  | MP | -**199.4** |  | MP | -69.9 +/- 15.1 |
|  |  |  |  | NSP | -94.1 |  | NSP | -89.3 +/- 11.2 |
| ßC1 | Rep | -136.4 | ßC1 | Rep | -48.6 | MP | Rep | -78.3 +/- 12.0 |
|  | TrAP | -83.2 |  | TrAP | -76.2 |  | TrAP | -68.4 +/- 13.3 |
|  | REn | -36.6 |  | REn | -141.1 |  | REn | -14.7 +/- 7.7 |
|  | C4 | -68 |  | C4 | -65.8 |  | C4 | -113.8 +/- 6.5 |
|  | CP | -78 |  | CP | -62.9 |  | CP | -103.3 +/- 5.6 |
|  | V2 | -61.6 |  | AV2 | -75.7 |  | AV2 | -77.1 +/- 13.8 |
|  | ßC1 | -47.9 |  | MP | **-199.7** |  | MP | -62.1 +/- 4.0 |
|  |  |  |  | NSP | **-96.3** |  | NSP | -74.7 +/- 4.2 |
|  |  |  |  |  |  | NSP | Rep | -74.4 +/- 16.3 |
|  |  |  |  |  |  |  | TrAP | -82.8 +/- 12.5 |
|  |  |  |  |  |  |  | REn | -22.2 +/- 16.8 |
|  |  |  |  |  |  |  | C4 | -133.9 +/- 7.8 |
|  |  |  |  |  |  |  | CP | -82.0 +/- 5.4 |
|  |  |  |  |  |  |  | AV2 | -81.3 +/- 9.8 |
|  |  |  |  |  |  |  | MP | -69.0 +/- 9.2 |
|  |  |  |  |  |  |  | NSP | -110.1 +/- 2.3 |
