# Supplementary material for: In silico evaluation of molecular virus–virus interactions taking place between Cotton leaf curl Kokhran virus- Burewala strain and Tomato leaf curl New Delhi virus
Source: PeerJ. 2021 Oct 19;9:e12018. doi: 10.7717/peerj.12018 (PMC8532979; doi:10.7717/peerj.12018)
Supplement: Supplemental Information 5 [file peerj-09-12018-s005.docx]

Binding Affinity ΔG (kcal mol-1) and the Dissociation Constant K_d_ (M) at 25.0 for the interactions observed between the proteins of CLCuKoV-Bur and ToLCNDV calculated through PRODIGY server

| **CLCuKoV-Bu** | **ToLCNDV** | **ΔG (kcal mol^-1^)** | **K_d_ (M) at 25.0 ℃** |
| --- | --- | --- | --- |
| **Rep** | Rep | -16.5 | 8.3E-13 |
|  | TrAP | -14.9 | 1.3E-11 |
|  | REn | -14.0 | 5.7E-11 |
|  | AC4 | -11.9 | 1.8E-09 |
|  | CP | -12.3 | 9.6E-10 |
|  | AV2 | -10.3 | 2.7E-08 |
|  | MP | -14.0 | 5.6E-11 |
|  | NSP | -16.4 | 9.8E-13 |
| **TrAP** | Rep | -15.8 | 2.7E-12 |
|  | TrAP | -14.2 | 3.6E-11 |
|  | REn | -16.1 | 1.6E-12 |
|  | AC4 | -12.0 | 1.7E-09 |
|  | CP | -16.1 | 1.6e-12 |
|  | AV2 | -12.6 | 6.0E-10 |
|  | MP | -13.5 | 1.4E-10 |
|  | NSP | -14.6 | 2.0E-11 |
| **REn** | Rep | -12.4 | 8.2E-10 |
|  | TrAP | -14.9 | 1.1E-11 |
|  | REn | -11.2 | 6.2E-09 |
|  | AC4 | -10.8 | 1.1E-08 |
|  | CP | -14.7 | 1.7E-11 |
|  | AV2 | -10.9 | 9.9E-09 |
|  | MP | -13.4 | 1.5E-10 |
|  | NSP | -11.5 | 3.5E-09 |
| **C4** | Rep | -15.8 | 2.7E-12 |
|  | TrAP | -14.2 | 3.6E-11 |
|  | REn | -16.8 | 4.7E-13 |
|  | AC4 | -14.7 | 1.6E-11 |
|  | CP | -10.7 | 1.4E-08 |
|  | AV2 | -14.4 | 2.6E-11 |
|  | MP | -12.6 | 6.0e-10 |
|  | NSP | -13.5 | 1.4E-10 |
| **CP** | Rep | -15.8 | 2.4E-12 |
|  | TrAP | -17.7 | 1.0E-13 |
|  | REn | -12.7 | 4.9E-10 |
|  | AC4 | -15.6 | 3.6E-12 |
|  | CP | -15.1 | 8.1E-12 |
|  | AV2 | -11.2 | 6.1E-09 |
|  | MP | -14.3 | 3.4E-11 |
|  | NSP | -12.7 | 4.8E-10 |
| **V2** | Rep | -12.2 | 1.0E-09 |
|  | TrAP | -19.0 | 1.1E-14 |
|  | REn | -15 | 1.0E-11 |
|  | AC4 | -11.7 | 2.8E-09 |
|  | CP | -13.2 | 2.0E-10 |
|  | AV2 | -11.1 | 6.8E-09 |
|  | MP | -15.2 | 7.3E-12 |
|  | NSP | -11.6 | 3.2E-09 |
| **CLCuMuB**  **βC1** | Rep | -9.3 | 1.6E-07 |
|  | TrAP | -15.7 | 2.8E-12 |
|  | REn | -12.7 | 5.0E-10 |
|  | AC4 | -14.1 | 4.8E-11 |
|  | CP | -12.2 | 1.1E-09 |
|  | AV2 | -11.4 | 4.1E-09 |
|  | MP | -15.8 | 2.4E-12 |
|  | NSP | -10.8 | 1.2E-08 |
| **CLCuKoV-Bu** | **CLCuKoV-Bu** | **ΔG (kcal mol^-1^)** | **K_d_ (M) at 25.0 ℃** |
| **Rep** | Rep | -14.4 | 3.0E-11 |
|  | TrAP | -12.1 | 1.4E-09 |
|  | REn | -12.9 | 3.6E-10 |
|  | C4 | -14.7 | 1.7E-11 |
|  | CP | -11.5 | 3.6E-09 |
|  | V2 | -13.5 | 1.2E-10 |
|  | βC1 | -11.5 | 3.6E-09 |
| **TrAP** | Rep | -9.5 | 1.0E-07 |
|  | TrAP | -10.7 | 1.5E-08 |
|  | REn | -10.8 | 1.3E-08 |
|  | C4 | -12.9 | 3.4E-10 |
|  | CP | -11.9 | 1.8E-09 |
|  | V2 | -12.0 | 1.5E-09 |
|  | βC1 | -12.4 | 7.5E-10 |
| **REn** | Rep | -12.9 | 3.6E-10 |
|  | TrAP | -10.8 | 1.3E-08 |
|  | REn | -14.3 | 3.1E-11 |
|  | C4 | -13.7 | 8.6E-11 |
|  | CP | -14.1 | 4.6E-11 |
|  | V2 | -10.7 | 1.3E08 |
|  | βC1 | -10.4 | 2.5E-08 |
| **C4** | Rep | -14.7 | 1.7E-11 |
|  | TrAP | -12.9 | 3.4E-10 |
|  | REn | -13.7 | 8.6E-11 |
|  | C4 | -10.5 | 2.1E-08 |
|  | CP | -13.0 | 2.7E-10 |
|  | V2 | -12.8 | 4.1E10 |
|  | βC1 | -12.3 | 9.6E-10 |
| **CP** | Rep | -11.5 | 3.6E-09 |
|  | TrAP | -11.9 | 1.8E-09 |
|  | REn | -14.1 | 4.6E-11 |
|  | C4 | -13.0 | 2.7E-10 |
|  | CP | -10.8 | 1.2E-08 |
|  | V2 | -10.7 | 1.5E-08 |
|  | βC1 | -12.5 | 6.8E-10 |
| **V2** | Rep | -13.5 | 1.2E-10 |
|  | TrAP | -12.0 | 1.5E-09 |
|  | REn | -10.7 | 1.3E08 |
|  | C4 | -12.8 | 4.1E10 |
|  | CP | -10.7 | 1.5E-08 |
|  | V2 | -10.3 | 2.8E-08 |
|  | βC1 | -10.2 | 3.1E-08 |
| **CLCuMuB**  **βC1** | Rep | -11.5 | 3.6E-09 |
|  | TrAP | -12.4 | 7.5E-10 |
|  | REn | -10.4 | 2.5E-08 |
|  | C4 | -12.3 | 9.6E-10 |
|  | CP | -12.5 | 6.8E-10 |
|  | V2 | -10.2 | 3.1E-08 |
|  | βC1 | -8.9 | 2.9E-07 |
| **ToLCNDV** | **ToLCNDV** | **ΔG (kcal mol^-1^)** | **K_d_ (M) at 25.0 ℃** |
| **Rep** | Rep | -16.6 | 7.2E-13 |
|  | TrAP | -16.3 | 1.1E-12 |
|  | REn | -14.9 | 1.2E-11 |
|  | AC4 | -11.9 | 2.0E-09 |
|  | CP | -14.1 | 4.6E-11 |
|  | AV2 | -14.6 | 1.8E-11 |
|  | MP | -11.3 | 4.8E-09 |
|  | NSP | -10 | 4.3E-08 |
| **TrAP** | Rep | -16.3 | 1.1E-12 |
|  | TrAP | -14.6 | 1.9E-11 |
|  | REn | -16.0 | 2.0E-12 |
|  | AC4 | -12.8 | 3.8E-10 |
|  | CP | -20.9 | 4.8E-16 |
|  | AV2 | -13.9 | 6.6E-11 |
|  | MP | -13.5 | 1.3E-10 |
|  | NSP | -10.3 | 2.7E-08 |
| **REn** | Rep | -14.9 | 1.2E-11 |
|  | TrAP | -16.0 | 2.0E-12 |
|  | REn | -12.9 | 3.3E-10 |
|  | AC4 | -16.1 | 1.5E-12 |
|  | CP | -13.9 | 6.6E-11 |
|  | AV2 | -14.5 | 2.2E-11 |
|  | MP | -11.4 | 4.2E-09 |
|  | NSP | -11.0 | 8.1E-09 |
| **AC4** | Rep | -11.9 | 2.0E-09 |
|  | TrAP | -12.8 | 3.8E-10 |
|  | REn | -16.1 | 1.5E-12 |
|  | AC4 | -12.8 | 3.9E-10 |
|  | CP | -17.5 | 1.5E-13 |
|  | AV2 | -14.2 | 4.1E-11 |
|  | MP | -15.3 | 5.6E-12 |
|  | NSP | -11.5 | 3.5E-09 |
| **CP** | Rep | -14.1 | 4.6E-11 |
|  | TrAP | -20.9 | 4.8E-16 |
|  | REn | -13.9 | 6.6E-11 |
|  | AC4 | -17.5 | 1.5E-13 |
|  | CP | -14.7 | 1.7E-11 |
|  | AV2 | -13.1 | 2.5E-10 |
|  | MP | -12.8 | 4.0E-10 |
|  | NSP | -12.3 | 9.0E-10 |
| **AV2** | Rep | -14.6 | 1.8E-11 |
|  | TrAP | -13.9 | 6.6E-11 |
|  | REn | -14.5 | 2.2E-11 |
|  | AC4 | -14.2 | 4.1E-11 |
|  | CP | -13.1 | 2.5E-10 |
|  | AV2 | -15.1 | 8.3E-12 |
|  | MP | -10.9 | 1.0E-08 |
|  | NSP | -10.4 | 2.4E-08 |
| **MP** | Rep | -11.3 | 4.8E-09 |
|  | TrAP | -13.5 | 1.3E-10 |
|  | REn | -11.4 | 4.2E-09 |
|  | AC4 | -15.3 | 5.6E-12 |
|  | CP | -12.8 | 4.0E-10 |
|  | AV2 | -10.9 | 1.0E-08 |
|  | MP | -10.3 | 3.0E08 |
|  | NSP | -8.5 | 6.0E-07 |
| **NSP** | Rep | -10 | 4.3E-08 |
|  | TrAP | -10.3 | 2.7E-08 |
|  | REn | -11.0 | 8.1E-09 |
|  | AC4 | -11.5 | 3.5E-09 |
|  | CP | -12.3 | 9.0E-10 |
|  | AV2 | -15.1 | 8.3E-12 |
|  | MP | -8.5 | 6.0E-07 |
|  | NSP | -7.8 | 2.0E-06 |
