# Supplementary material for: In silico evaluation of molecular virus–virus interactions taking place between Cotton leaf curl Kokhran virus- Burewala strain and Tomato leaf curl New Delhi virus
Source: PeerJ. 2021 Oct 19;9:e12018. doi: 10.7717/peerj.12018 (PMC8532979; doi:10.7717/peerj.12018)
Supplement: Supplemental Information 6 [file peerj-09-12018-s006.docx]

**Number of Interfacial contacts (ICs) and Non Interacting Surface per property (NIS) among the protein-protein interactions performed**

| **CLCuKoV-Bu** | **ToLCNDV** | **Number of Interfacial Contacts (ICs) per property** | | | | | | **Non Interacting Surface (NIS) per property:** | |
| --- | --- | --- | --- | --- | --- | --- | --- | --- | --- |
|  |  | **ICs charged-charged** | **ICs charged-polar:** | **ICs charged-apolar:** | **ICs polar-polar:** | **ICs polar-apolar:** | **ICs apolar-apolar:** | **NIS charged:** | **NIS Apolar:** |
| **Rep** | Rep | 18 | 20 | 40 | 4 | 30 | 31 | 24.96% | 41.50% |
|  | TrAP | 13 | 29 | 41 | 12 | 27 | 19 | 25% | 39.58 |
|  | REn | 12 | 23 | 36 | 10 | 28 | 24 | 22.89% | 37.78 |
|  | AC4 | 10 | 19 | 14 | 13 | 29 | 11 | 24.05% | 37.84% |
|  | CP | 4 | 16 | 27 | 5 | 22 | 40 | 26.05% | 40.53% |
|  | AV2 | 12 | 26 | 37 | 13 | 12 | 14 | 24.82% | 38.57% |
|  | MP | 9 | 27 | 42 | 18 | 29 | 20 | 24.67% | 35.7& |
|  | NSP | 17 | 21 | 33 | 16 | 42 | 30 | 24.46% | 40.11% |
| **TrAP** | Rep | 9 | 13 | 33 | 7 | 36 | 19 | 23.79% | 41.89% |
|  | TrAP | 15 | 21 | 37 | 7 | 33 | 19 | 21.36% | 43.64% |
|  | REn | 4 | 18 | 14 | 10 | 34 | 30 | 19.20% | 35.20% |
|  | AC4 | 8 | 22 | 23 | 23 | 35 | 10 | 18.59% | 42.31% |
|  | CP | 12 | 15 | 32 | 4 | 31 | 25 | 24.17% | 38.07% |
|  | AV2 | 10 | 35 | 29 | 18 | 29 | 16 | 22.38% | 33.72% |
|  | MP | 9 | 11 | 31 | 4 | 26 | 26 | 22.71% | 39.06% |
|  | NSP | 9 | 11 | 31 | 4 | 26 | 26 | 22.71% | 39.06% |
| **REn** | Rep | 15 | 12 | 40 | 2 | 13 | 9 | 26.58% | 42.11% |
|  | TrAP | 10 | 21 | 40 | 5 | 10 | 9 | 30.94% | 35.97% |
|  | REn | 9 | 25 | 27 | 10 | 28 | 15 | 22.50% | 31.25% |
|  | AC4 | 12 | 26 | 15 | 15 | 19 | 7 | 27.38% | 28.57% |
|  | CP | 14 | 15 | 36 | 1 | 21 | 14 | 28.27% | 36.71% |
|  | AV2 | 9 | 30 | 19 | 7 | 16 | 5 | 28.68% | 32.56% |
|  | MP | 9 | 22 | 24 | 9 | 25 | 10 | 27% | 31.69% |
|  | NSP | 5 | 23 | 20 | 7 | 24 | 15 | 25% | 39.47% |
| **C4** | Rep | 9 | 13 | 33 | 7 | 36 | 19 | 23.79% | 41.89% |
|  | TrAP | 4 | 18 | 14 | 10 | 34 | 30 | 19.205 | 35.20% |
|  | REn | 7 | 14 | 35 | 6 | 36 | 30 | 23.91% | 37.39% |
|  | AC4 | 6 | 16 | 18 | 9 | 33 | 16 | 19.88% | 35.09% |
|  | CP | 7 | 7 | 30 | 3 | 25 | 20 | 24.04% | 37.09% |
|  | AV2 | 15 | 23 | 11 | 8 | 25 | 14 | 22.17% | 37.10% |
|  | MP | 10 | 35 | 29 | 18 | 29 | 16 | 22.38% | 33.72% |
|  | NSP | 9 | 11 | 31 | 4 | 26 | 26 | 22.71% | 39.06% |
| **CP** | Rep | 19 | 22 | 47 | 6 | 26 | 22 | 26.84% | 40.81% |
|  | TrAP | 13 | 8 | 23 | 3 | 21 | 23 | 28.30% | 37.74% |
|  | REn | 11 | 24 | 41 | 14 | 41 | 33 | 25.31% | 34.38% |
|  | AC4 | 6 | 13 | 22 | 8 | 39 | 14 | 27.67% | 35.18% |
|  | CP | 14 | 18 | 45 | 5 | 23 | 36 | 27.91% | 37.62% |
|  | AV2 | 9 | 13 | 16 | 4 | 18 | 15 | 28.62% | 35.02% |
|  | MP | 17 | 19 | 33 | 7 | 21 | 22 | 26.48% | 33.81% |
|  | NSP | 8 | 12 | 34 | 3 | 18 | 27 | 26.30% | 38.80% |
| **V2** | Rep | 13 | 12 | 36 | 1 | 13 | 18 | 25.52% | 41.53% |
|  | TrAP | 14 | 16 | 30 | 8 | 31 | 16 | 26.5% | 38% |
|  | REn | 9 | 22 | 35 | 10 | 45 | 28 | 21.72% | 34.84% |
|  | AC4 | 8 | 23 | 17 | 5 | 17 | 9 | 23.61% | 34.03% |
|  | CP | 11 | 10 | 42 | 3 | 15 | 26 | 27.24% | 37.54% |
|  | AV2 | 13 | 25 | 25 | 3 | 10 | 15 | 25.13% | 36.13% |
|  | MP | 18 | 27 | 33 | 7 | 23 | 24 | 24.19% | 33.55% |
|  | NSP | 12 | 18 | 26 | 5 | 16 | 19 | 25.37% | 38.81% |
| **CLCuMuB**  **βC1** | Rep | 9 | 10 | 27 | 0 | 8 | 23 | 26.29% | 44.94% |
|  | TrAP | 10 | 4 | 31 | 5 | 25 | 27 | 27.625 | 43.81% |
|  | REn | 6 | 14 | 25 | 13 | 43 | 32 | 23.42% | 38.74% |
|  | AC4 | 6 | 9 | 14 | 4 | 37 | 21 | 26.90% | 41.38% |
|  | CP | 10 | 6 | 39 | 2 | 14 | 30 | 27.72% | 40.26% |
|  | AV2 | 13 | 9 | 20 | 2 | 18 | 25 | 26.60% | 41.49% |
|  | MP | 8 | 19 | 36 | 8 | 34 | 40 | 25.41% | 37.95% |
|  | NSP | 4 | 8 | 24 | 4 | 19 | 28 | 25.68% | 42.30% |
| **CLCuKoV-Bu** | **CLCuKoV-Bu** | **Number of Interfacial Contacts (ICs) per property** | | | | | | **Non-Interacting Surface (NIS) per property:** | |
|  |  | **ICs charged-charged** | **ICs charged-polar:** | **ICs charged-apolar:** | **ICs polar-polar:** | **ICs polar-apolar:** | **ICs apolar-apolar:** | **NIS charged:** | **NIS Apolar:** |
| **Rep** | Rep | 20 | 26 | 32 | 5 | 24 | 27 | 25% | 40.96% |
|  | TrAP | 17 | 14 | 26 | 10 | 23 | 11 | 27% | 40.77% |
|  | REn | 8 | 15 | 32 | 8 | 25 | 27 | 22.7% | 42.7% |
|  | C4 | 9 | 24 | 24 | 4 | 31 | 18 | 23.4% | 40.2% |
|  | CP | 5 | 8 | 22 | 4 | 23 | 37 | 26.2% | 42% |
|  | V2 | 19 | 5 | 42 | 1 | 12 | 16 | 25% | 39.7% |
|  | **βC1** | 5 | 8 | 22 | 4 | 23 | 37 | 26.2% | 42% |
| **TrAP** | Rep | 7 | 9 | 14 | 8 | 19 | 17 | 27% | 39.8% |
|  | TrAP | 19 | 15 | 12 | 8 | 16 | 3 | 40% | 25.7% |
|  | REn | 18 | 19 | 26 | 12 | 16 | 4 | 23.2% | 40.4% |
|  | C4 | 13 | 29 | 26 | 9 | 20 | 5 | 24.3% | 33.5% |
|  | CP | 12 | 16 | 24 | 5 | 18 | 9 | 30.6% | 34.4% |
|  | V2 | 9 | 16 | 24 | 2 | 15 | 6 | 30.4% | 32% |
|  | **βC1** | 15 | 9 | 22 | 6 | 27 | 10 | 34.4% | 39.2% |
| **REn** | Rep | 8 | 15 | 32 | 8 | 25 | 27 | 22.7% | 42.7% |
|  | TrAP | 18 | 19 | 26 | 12 | 16 | 4 | 23.2% | 40.4% |
|  | REn | 8 | 18 | 21 | 17 | 43 | 26 | 18.6% | 44.5% |
|  | C4 | 7 | 31 | 24 | 6 | 28 | 10 | 18.7% | 42.1% |
|  | CP | 20 | 23 | 37 | 6 | 19 | 18 | 24.2% | 38.7% |
|  | V2 | 10 | 27 | 26 | 10 | 18 | 9 | 21.0% | 42.6% |
|  | **βC1** | 6 | 18 | 23 | 6 | 20 | 21 | 23.7% | 45.6% |
| **C4** | Rep | 9 | 24 | 24 | 4 | 31 | 18 | 23.4% | 40.2% |
|  | TrAP | 13 | 29 | 26 | 9 | 20 | 5 | 24.3% | 33.5% |
|  | REn | 7 | 31 | 24 | 6 | 28 | 10 | 18.7% | 42.1% |
|  | C4 | 5 | 13 | 20 | 10 | 16 | 12 | 20.2% | 36.5% |
|  | CP | 12 | 9 | 31 | 6 | 19 | 26 | 25.2% | 36.3% |
|  | V2 | 11 | 11 | 29 | 2 | 13 | 13 | 22.4% | 35% |
|  | **βC1** | 8 | 3 | 15 | 4 | 25 | 20 | 24.5% | 39.6% |
| **CP** | Rep | 5 | 8 | 22 | 4 | 23 | 37 | 26.2% | 42% |
|  | TrAP | 3 | 8 | 24 | 4 | 18 | 14 | 32.1% | 32.6% |
|  | REn | 20 | 23 | 37 | 6 | 19 | 18 | 24.2% | 38.7% |
|  | C4 | 12 | 9 | 31 | 6 | 19 | 26 | 25.2% | 36.3% |
|  | CP | 2 | 4 | 14 | 3 | 20 | 29 | 29.7% | 35.2% |
|  | V2 | 6 | 9 | 31 | 1 | 8 | 17 | 28.8% | 35.2% |
|  | **βC1** | 9 | 10 | 42 | 2 | 14 | 29 | 29.7% | 38.4% |
| **V2** | Rep | 19 | 5 | 42 | 1 | 12 | 16 | 25% | 39.7% |
|  | TrAP | 9 | 16 | 24 | 2 | 15 | 6 | 30.4% | 32% |
|  | REn | 10 | 27 | 26 | 10 | 18 | 9 | 21.0% | 42.6% |
|  | C4 | 11 | 11 | 29 | 2 | 13 | 13 | 22.4% | 35% |
|  | CP | 6 | 9 | 31 | 1 | 8 | 17 | 28.8% | 35.2% |
|  | V2 | 17 | 10 | 19 | 0 | 5 | 13 | 25.5% | 36.1% |
|  | **βC1** | 6 | 6 | 34 | 2 | 11 | 20 | 27.6% | 42.5% |
| **CLCuMuB**  **βC1** | Rep | 5 | 8 | 22 | 4 | 23 | 37 | 26.2% | 42% |
|  | TrAP | 15 | 9 | 22 | 6 | 27 | 10 | 34.4% | 39.2% |
|  | REn | 6 | 18 | 23 | 6 | 20 | 21 | 23.7% | 45.6% |
|  | C4 | 8 | 3 | 15 | 4 | 25 | 20 | 24.5% | 39.6% |
|  | CP | 9 | 10 | 42 | 2 | 14 | 29 | 29.7% | 38.4% |
|  | V2 | 6 | 6 | 34 | 2 | 11 | 20 | 27.6% | 42.5% |
|  | **βC1** | 7 | 5 | 14 | 1 | 17 | 43 | 29.8% | 46.2% |

| **ToLCNDV** | **ToLCNDV** | **Number of Interfacial Contacts (ICs) per property** | | | | | | **Non Interacting Surface (NIS) per property:** | |
| --- | --- | --- | --- | --- | --- | --- | --- | --- | --- |
|  |  | **ICs charged-charged** | **ICs charged-polar:** | **ICs charged-apolar:** | **ICs polar-polar:** | **ICs polar-apolar:** | **ICs apolar-apolar:** | **NIS charged:** | **NIS Apolar:** |
| **Rep** | Rep | 13 | 26 | 50 | 5 | 31 | 31 | 25.5% | 43.6% |
|  | TrAP | 8 | 27 | 40 | 7 | 35 | 21 | 23.4% | 41.3% |
|  | REn | 17 | 19 | 44 | 3 | 22 | 31 | 25.3% | 42.65 |
|  | AC4 | 12 | 24 | 24 | 6 | 21 | 12 | 25% | 41.5% |
|  | CP | 12 | 21 | 40 | 3 | 22 | 36 | 25.7% | 41.9% |
|  | AV2 | 20 | 19 | 38 | 5 | 23 | 28 | 24.8% | 41.7% |
|  | MP | 16 | 14 | 33 | 7 | 17 | 19 | 25.8% | 44.6% |
|  | NSP | 16 | 9 | 30 | 4 | 8 | 15 | 28.1% | 40.4% |
| **TrAP** | Rep | 8 | 27 | 40 | 7 | 35 | 21 | 23.4% | 41.3% |
|  | TrAP | 3 | 17 | 23 | 18 | 39 | 25 | 18.9% | 35.4% |
|  | REn | 8 | 15 | 37 | 12 | 35 | 29 | 21.7% | 37.6% |
|  | AC4 | 6 | 24 | 21 | 26 | 37 | 16 | 19.1% | 34.4% |
|  | CP | 7 | 20 | 40 | 7 | 51 | 25 | 25.4% | 35.7% |
|  | AV2 | 8 | 15 | 26 | 14 | 29 | 25 | 20.7% | 34.2% |
|  | MP | 4 | 21 | 37 | 12 | 29 | 35 | 22.6% | 41% |
|  | NSP | 6 | 24 | 26 | 11 | 13 | 18 | 23..9% | 33.7% |
| **REn** | Rep | 17 | 19 | 44 | 3 | 22 | 31 | 25.3% | 42.65 |
|  | TrAP | 8 | 15 | 37 | 12 | 35 | 29 | 21.7% | 37.6% |
|  | REn | 20 | 25 | 41 | 6 | 15 | 29 | 25.4% | 41.3% |
|  | AC4 | 8 | 23 | 36 | 10 | 36 | 21 | 22.9% | 38.5% |
|  | CP | 11 | 13 | 34 | 3 | 21 | 29 | 27.3% | 36.9% |
|  | AV2 | 14 | 27 | 31 | 5 | 24 | 18 | 24.7% | 36.8% |
|  | MP | 9 | 12 | 37 | 5 | 17 | 21 | 27.3% | 43.7% |
|  | NSP | 17 | 12 | 26 | 3 | 11 | 9 | 31.5% | 35.6% |
| **AC4** | Rep | 12 | 24 | 24 | 6 | 21 | 12 | 25% | 41.5% |
|  | TrAP | 6 | 24 | 21 | 26 | 37 | 16 | 19.1% | 34.4% |
|  | REn | 8 | 23 | 36 | 10 | 36 | 21 | 22.9% | 38.5% |
|  | AC4 | 11 | 23 | 18 | 28 | 37 | 22 | 18.1% | 34.0% |
|  | CP | 16 | 24 | 44 | 8 | 34 | 25 | 23.6% | 38.7% |
|  | AV2 | 13 | 24 | 22 | 12 | 30 | 15 | 21% | 36.1% |
|  | MP | 9 | 13 | 21 | 5 | 38 | 9 | 25% | 41.4% |
|  | NSP | 17 | 18 | 17 | 10 | 18 | 7 | 29.3% | 31.0% |
| **CP** | Rep | 12 | 21 | 40 | 3 | 22 | 36 | 25.7% | 41.9% |
|  | TrAP | 7 | 20 | 40 | 7 | 51 | 25 | 25.4% | 35.7% |
|  | REn | 11 | 13 | 34 | 3 | 21 | 29 | 27.3% | 36.9% |
|  | AC4 | 16 | 24 | 44 | 8 | 34 | 25 | 23.6% | 38.7% |
|  | CP | 15 | 14 | 25 | 0 | 24 | 50 | 26.8% | 36.9% |
|  | AV2 | 9 | 12 | 33 | 7 | 21 | 32 | 26.6% | 36.0% |
|  | MP | 13 | 15 | 41 | 2 | 14 | 27 | 27.7% | 39.7% |
|  | NSP | 12 | 14 | 31 | 1 | 13 | 25 | 30.5% | 34.12% |
| **AV2** | Rep | 20 | 19 | 38 | 5 | 23 | 28 | 24.8% | 41.7% |
|  | TrAP | 8 | 15 | 26 | 14 | 29 | 25 | 20.7% | 34.2% |
|  | REn | 14 | 27 | 31 | 5 | 24 | 18 | 24.7% | 36.8% |
|  | AC4 | 13 | 24 | 22 | 12 | 30 | 15 | 21% | 36.1% |
|  | CP | 9 | 12 | 33 | 7 | 21 | 32 | 26.6% | 36.0% |
|  | AV2 | 8 | 24 | 23 | 2 | 28 | 25 | 25.2% | 34.0% |
|  | MP | 4 | 15 | 27 | 6 | 21 | 20 | 27.3% | 42.4% |
|  | NSP | 8 | 9 | 11 | 5 | 18 | 9 | 31.6% | 32.9% |
| **MP** | Rep | 16 | 14 | 33 | 7 | 17 | 19 | 25.8% | 44.6% |
|  | TrAP | 4 | 21 | 37 | 12 | 29 | 35 | 22.6% | 41% |
|  | REn | 9 | 12 | 37 | 5 | 17 | 21 | 27.3% | 43.7% |
|  | AC4 | 9 | 13 | 21 | 5 | 38 | 9 | 25% | 41.4% |
|  | CP | 13 | 15 | 41 | 2 | 14 | 27 | 27.7% | 39.7% |
|  | AV2 | 4 | 15 | 27 | 6 | 21 | 20 | 27.3% | 42.4% |
|  | MP | 10 | 5 | 19 | 6 | 24 | 22 | 30.8% | 45.7% |
|  | NSP | 3 | 7 | 19 | 2 | 12 | 13 | 34.2% | 38.8% |
| **NSP** | Rep | 16 | 9 | 30 | 4 | 8 | 15 | 28.1% | 40.4% |
|  | TrAP | 6 | 24 | 26 | 11 | 13 | 18 | 23..9% | 33.7% |
|  | REn | 17 | 12 | 26 | 3 | 11 | 9 | 31.5% | 35.6% |
|  | AC4 | 17 | 18 | 17 | 10 | 18 | 7 | 29.3% | 31.0% |
|  | CP | 12 | 14 | 31 | 1 | 13 | 25 | 30.5% | 34.12% |
|  | AV2 | 8 | 9 | 11 | 5 | 18 | 9 | 31.6% | 32.9% |
|  | MP | 3 | 7 | 19 | 2 | 12 | 13 | 34.2% | 38.8% |
|  | NSP | 8 | 5 | 15 | 2 | 3 | 21 | 41.8% | 26.5% |
